# Robotic-Assisted Resection of a Giant Mesenteric Lymphangioma in a 36-Year-Old Woman: A Case Report

**DOI:** 10.7759/cureus.107790

**Published:** 2026-04-27

**Authors:** Thalia Petropoulou, Kyriacos Evangelou, Andreas Polydorou

**Affiliations:** 1 Department of Robotic Colon and Rectal Surgery, University Hospitals Sussex NHS Foundation Trust, Brighton, GBR; 2 Second Department of Surgery, Aretaieion University Hospital, Athens, GRC; 3 Department of Minimally Invasive and Robotic Surgery, Athens Euroclinic, Athens, GRC; 4 School of Medicine, National and Kapodistrian University of Athens, Athens, GRC

**Keywords:** indocyanine green (icg), lymphatic malformation, mesenteric cystic lymphangioma, mesenteric lymphangioma, mesenteric tumor, robotic surgical procedures, small bowel tumor, surgical case reports

## Abstract

We report a case of a 36-year-old woman who presented with acute abdominal pain and repeated episodes of nonbilious vomiting. Contrast-enhanced computed tomography demonstrated a large, multiloculated cystic lesion measuring approximately 15 × 10 × 8 cm within the small bowel mesentery, without solid components or radiologic features suggestive of malignancy. Because of the patient’s symptoms, lesion size, and close relationship to mesenteric vessels, robotic-assisted resection was performed with adjunctive indocyanine green (ICG) fluorescence imaging to facilitate intraoperative vascular assessment and confirm bowel perfusion. Intraoperatively, a giant, translucent cystic mass arising from the jejunal mesentery was identified and completely excised without rupture or bowel resection. Histopathologic examination showed dilated lymphatic spaces lined by flattened endothelial cells within fibrous septa containing lymphoid aggregates, consistent with cystic lymphangioma. The postoperative course was uneventful, and the patient was discharged on the first postoperative day. At the six-month follow-up, she remained asymptomatic with no evidence of recurrence on ultrasonography. This case supports robotic-assisted excision as a feasible, minimally invasive option for selected giant mesenteric lymphangiomas in adults.

## Introduction

Mesenteric lymphangiomas are rare benign lymphatic malformations that arise from abnormal development of lymphatic channels. Although lymphangiomas are most commonly diagnosed in the head, neck, and axilla during childhood, intra-abdominal involvement is uncommon, and adult presentation is particularly rare [1,2]. When they occur within the abdomen, the small bowel mesentery is among the most frequent sites. Clinical manifestations are often nonspecific and depend on lesion size, location, and the presence of complications such as hemorrhage, infection, volvulus, or bowel obstruction [1-4].

Cross-sectional imaging typically demonstrates a thin-walled, uni- or multiloculated cystic mesenteric lesion, but definitive diagnosis relies on histopathologic examination [1,3]. Complete surgical excision is generally recommended for symptomatic lesions and for lesions with uncertain preoperative diagnosis because incomplete resection may predispose to recurrence [1-3].

Recent case reports continue to highlight adult mesenteric lymphangioma and related mesenteric lymphatic malformations as rare but important causes of acute abdominal pathology [5-7]. Minimally invasive excision may be feasible in selected cases, and robotic surgery may be particularly useful when dissection near mesenteric vessels is required [8-10]. In addition, indocyanine green (ICG) fluorescence imaging has emerging applications in minimally invasive surgery for vascular assessment and tissue perfusion analysis [11,12]. However, reports describing robotic-assisted resection of giant mesenteric lymphangiomas in adults, particularly with adjunctive fluorescence guidance for vascular assessment and bowel perfusion, remain exceptionally limited. We therefore report a symptomatic giant mesenteric lymphangioma in a 36-year-old woman that was successfully managed with robotic-assisted resection and adjunctive ICG fluorescence imaging.

## Case presentation

A 36-year-old woman presented to the emergency department with a 24-hour history of diffuse colicky abdominal pain and multiple episodes of nonbilious vomiting. She reported progressive worsening of the pain and mild abdominal distension. There was no history of previous abdominal surgery, abdominal trauma, or chronic gastrointestinal disease. She denied hematemesis, melena, and recent changes in bowel habits.

On physical examination, the abdomen was moderately distended, with tenderness most prominent in the periumbilical region and right lower quadrant. No peritoneal signs were present. No palpable mass was appreciated because of discomfort and distension. Bowel sounds were present and mildly hypoactive. She was hemodynamically stable. Laboratory evaluation, including complete blood count, renal and liver function testing, serum amylase, serum lipase, and C-reactive protein, was unremarkable (Table 1).

**Table 1:** Admission vital signs and laboratory investigations.

Parameter	Result	Reference range
Vital signs		
Temperature	36.8 °C	36.1-37.2 °C
Heart rate	88 beats/min	60-100 beats/min
Blood pressure	118/74 mmHg	90-120/60-80 mmHg
Respiratory rate	16 breaths/min	12-20 breaths/min
Peripheral oxygen saturation on room air	99%	95-100%
Complete blood count		
Hemoglobin	12.8 g/dL	12.0-15.5 g/dL
Hematocrit	38.40%	36.0-46.0%
White blood cell count	7.4 ×10^9^/L	4.0-10.0 ×10^9^/L
Platelet count	281 ×10^9^/L	150-400 ×10^9^/L
Renal function/electrolytes		
Sodium	138 mmol/L	135-145 mmol/L
Potassium	3.9 mmol/L	3.5-5.1 mmol/L
Chloride	102 mmol/L	98-107 mmol/L
Bicarbonate	24 mmol/L	22-29 mmol/L
Blood urea nitrogen	13 mg/dL	7-20 mg/dL
Creatinine	0.78 mg/dL	0.50-0.95 mg/dL
Liver function tests		
Aspartate aminotransferase	21 U/L	10-35 U/L
Alanine aminotransferase	18 U/L	7-35 U/L
Alkaline phosphatase	79 U/L	35-104 U/L
Total bilirubin	0.6 mg/dL	0.2-1.2 mg/dL
Albumin	4.2 g/dL	3.5-5.0 g/dL
Other investigations		
Serum amylase	57 U/L	30-110 U/L
Serum lipase	31 U/L	13-60 U/L
C-reactive protein	2.0 mg/L	<5.0 mg/L

After initial clinical assessment and laboratory evaluation, contrast-enhanced computed tomography of the abdomen and pelvis was obtained during the same admission, and it showed a well-circumscribed multiloculated cystic lesion measuring approximately 15 × 10 × 8 cm arising from the small bowel mesentery. The lesion had thin, smooth walls without mural nodules, calcifications, or appreciable septal thickening. Adjacent small bowel loops were displaced and compressed, but there was no definite bowel obstruction, pneumoperitoneum, or free intraperitoneal fluid. The radiologic differential diagnosis included mesenteric cyst and mesenteric lymphangioma.

Given the patient’s symptoms, lesion size, and a close relationship of the mass to the mesenteric vessels, operative management was undertaken. Written informed consent was obtained for treatment and publication of case details and accompanying images.

**Figure 1:**
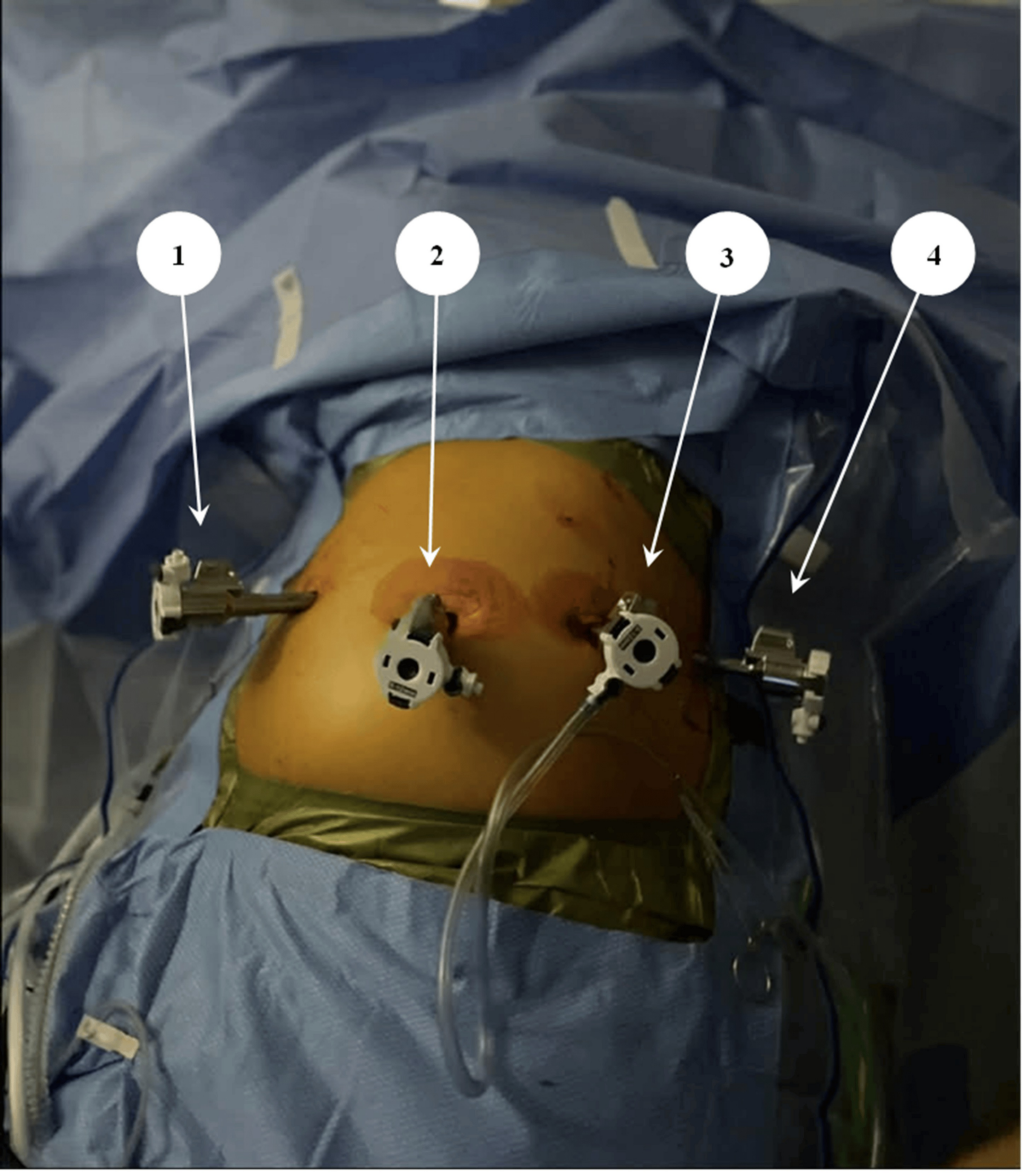
Port placement in smile-line configuration. Visible trocars include four 8-mm robotic ports: (1) left lateral robotic port, (2) umbilical camera port, (3) right paramedian robotic port, and (4) right lateral robotic port.

The procedure was performed using the da Vinci Xi robotic platform (Intuitive Surgical, Inc., Sunnyvale, USA). The patient was positioned supine with a slight Trendelenburg. Pneumoperitoneum was established at 12 mmHg. Four 8-mm robotic ports (Intuitive Surgical, Inc., Sunnyvale, USA) and one AirSeal® access/assistant port (CONMED Corporation, Utica, USA) were placed in a smile-line configuration targeting the mid-abdomen (Figure 1).

**Figure 2:**
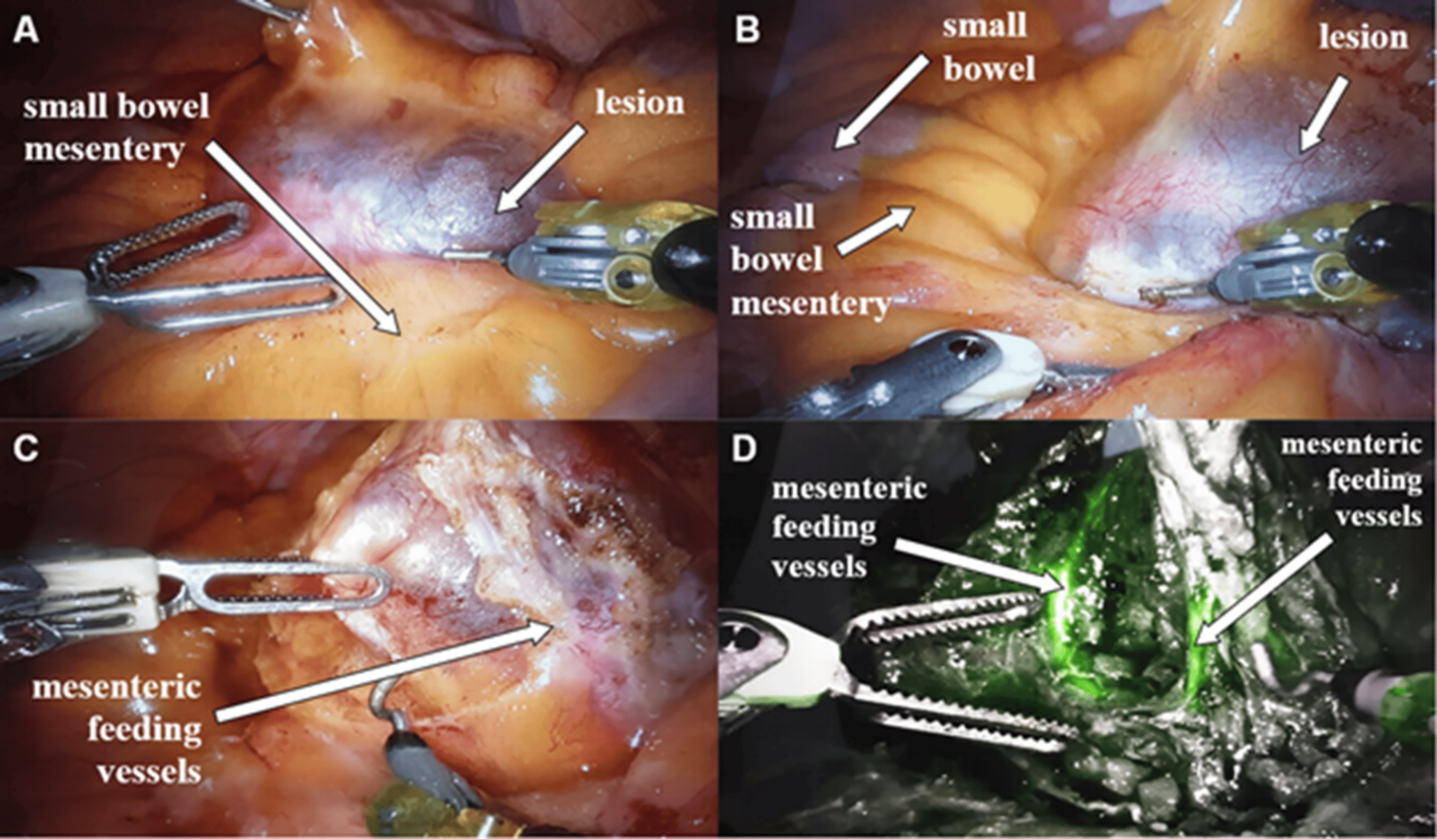
Intraoperative views from the robotic-assisted resection of a giant, multiloculated, translucent cystic lesion arising from the jejunal mesentery. (A) The lesion is seen as a smooth, thin-walled, translucent cystic mass expanding the mesentery, with a bluish-white sheen and fine surface vascular markings, as it is gently grasped and retracted to define its extent. (B) Dissection along the small-bowel mesentery demonstrates the mesenteric leaf stretched over the cyst and helps delineate the mesenteric plane and broad-based attachment of the lesion. (C) Progressive mesenteric dissection identifies and controls the mesenteric feeding vessels supplying the lesion, permitting safe separation while preserving the adjacent mesenteric vasculature. (D) Near-infrared indocyanine green fluorescence imaging is used as an adjunct during resection to enhance visualization of the mesenteric vascular anatomy, including the feeding vessels, and to guide safe vascular dissection.

After docking and systematic exploration, a giant, multiloculated, translucent cystic mass arising from the jejunal mesentery was identified, displacing adjacent bowel loops (Figure 2A).

No bowel infiltration was observed (Figure 2B), and the feeding mesenteric vessels were identified (Figure 2C). ICG was administered intraoperatively after identification of the lesion and mesenteric vascular pedicle. A total of 10 mL of reconstituted ICG solution (2.5 mg/mL; cumulative dose 25 mg) was given intravenously in two divided doses: first during mesenteric dissection to enhance vascular assessment and then after excision to confirm preserved bowel perfusion with near-infrared fluorescence imaging (Figure 2D).

Careful robotic sharp and blunt dissection with ProGrasp forceps (Intuitive Surgical, Inc., Sunnyvale, USA) and da Vinci permanent cautery hook (Intuitive Surgical, Inc., Sunnyvale, USA) allowed progressive mobilization of the lesion from the surrounding mesenteric fat and vessels. No major vascular encasement was identified. The lesion was completely excised intact without cyst rupture and without bowel resection. The specimen was placed in an endoscopic retrieval bag and removed through a small extension of the umbilical incision. No drain was placed. Total operative time was 140 minutes, and estimated blood loss was 20 mL.

**Figure 3:**
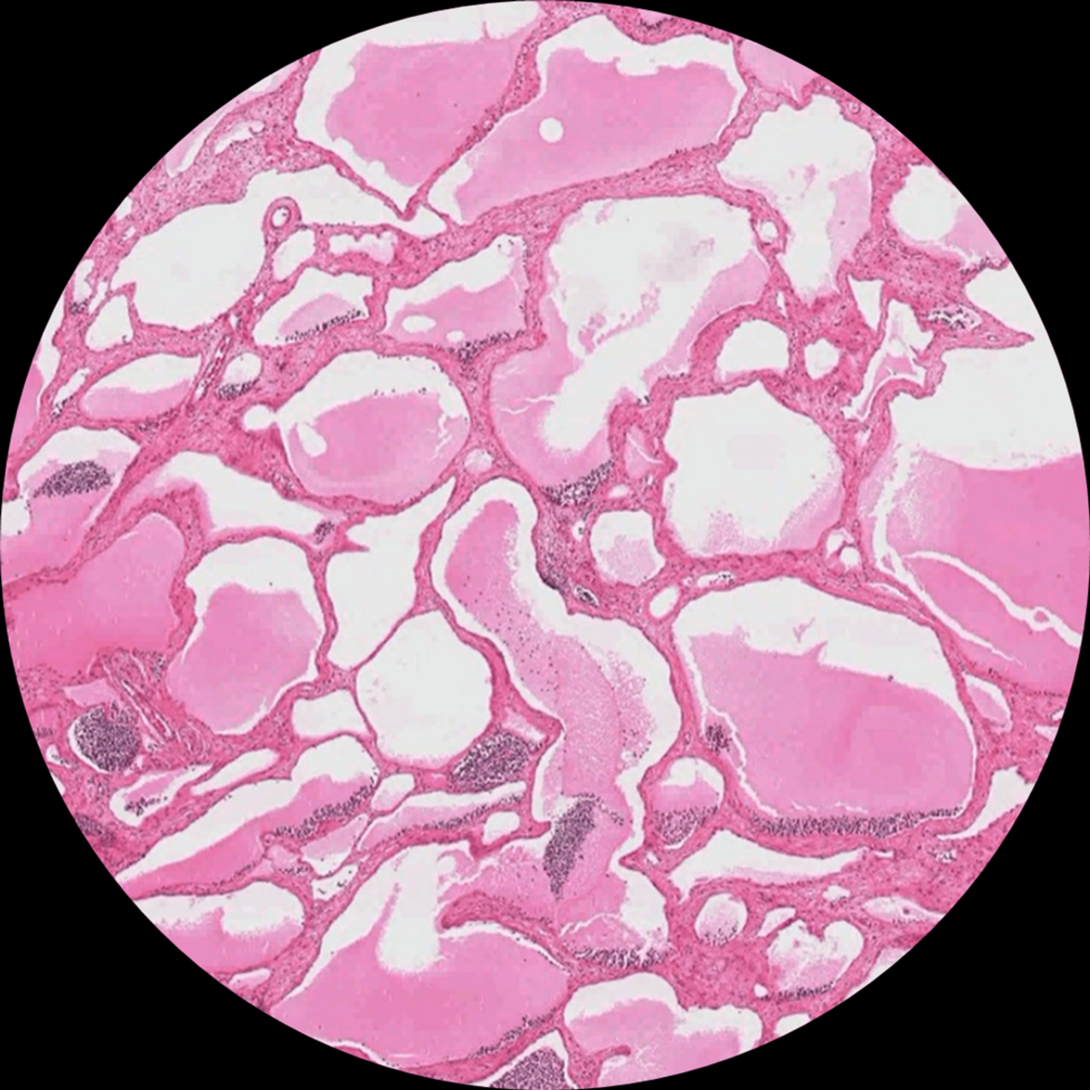
Lesion micrograph demonstrating features consistent with cystic lymphangioma (H&E stain, 10x magnification). Low-power view shows a multiloculated lesion composed of numerous variably sized, irregularly shaped cystic spaces separated by thin fibrous septa. Many spaces are optically empty, while others contain pale eosinophilic to more homogeneous proteinaceous material. Along several septa, there are small, dark basophilic lymphoid aggregates/foci of chronic inflammatory cells. The cyst walls appear thin, and the lining is considerably attenuated and flattened; no obvious solid epithelial proliferation, papillary architecture, marked cytologic atypia, brisk mitotic activity, or overt necrosis are recognized.

Gross examination demonstrated a multiloculated cystic lesion measuring 15 cm in maximum dimension with thin, translucent walls and clear serous fluid content. Microscopically, the lesion was composed of dilated lymphatic spaces lined by flattened endothelial cells within fibrous septa containing lymphoid aggregates and smooth muscle fibers (Figure 3).

No epithelial lining, cytologic atypia, or malignant features were identified. Resection margins were clear, consistent with an R0 excision. The findings were diagnostic of cystic mesenteric lymphangioma.

The postoperative course was uneventful. Oral intake was resumed on the day of surgery, and the patient was discharged on the first postoperative day. No postoperative complications, including bleeding, infection, or lymphatic leak, occurred. At six-month follow-up, she remained asymptomatic with full return to normal activities, and abdominal ultrasonography showed no residual lesion or recurrence.

## Discussion

Mesenteric lymphangiomas are uncommon in adults and can present either incidentally or with acute symptoms caused by mass effect and secondary complications [1-7]. In adult patients, abdominal pain, nausea, vomiting, distension, volvulus, and bowel obstruction have all been described. In the present case, the lesion presented with acute abdominal pain and vomiting related to compression of adjacent bowel loops, but without bowel necrosis or the need for intestinal resection.

Imaging is central to preoperative assessment, although it is rarely diagnostic on its own. On computed tomography, mesenteric lymphangiomas usually appear as well-defined cystic lesions with thin walls and internal septations, and imaging is particularly helpful in defining size, location, and relationships to the bowel and mesenteric vessels [3]. Histopathologic evaluation remains necessary for definitive diagnosis and for exclusion of other cystic mesenteric lesions.

Complete excision remains the treatment of choice for symptomatic lesions [1-3]. Organ-preserving enucleation is feasible when the lesion can be separated from the bowel and the mesenteric blood supply, whereas bowel resection may be required when there is inseparable vascular involvement, bowel wall infiltration, or ischemic compromise [4-8]. In our patient, the lesion was successfully dissected from adjacent mesenteric vessels, allowing complete R0 excision without intestinal sacrifice.

The literature on adult mesenteric lymphangioma remains dominated by open and conventional laparoscopic cases [5-8]. Robotic experience is far more limited and is mostly described for other mesenteric cystic lesions or pediatric series [9,10]. In selected patients, the robotic platform may facilitate meticulous dissection by providing stable three-dimensional visualization and articulated instrumentation [13-15], which can be useful in confined mesenteric planes. In the present case, these technical advantages supported precise dissection, minimal blood loss, intact specimen retrieval, and rapid postoperative recovery.

ICG fluorescence imaging is increasingly used in minimally invasive surgery for tissue perfusion assessment and vascular visualization [11,12]. In our case, it was used as an adjunct during resection to support safe dissection and confirm preserved bowel perfusion after excision. Although the role of fluorescence guidance in benign mesenteric lymphatic lesions is not yet standardized, this case suggests that it may be a helpful complement during complex minimally invasive mesenteric surgery. In adults, recommended intravenous doses for visualization of vessels, blood flow, and tissue perfusion are typically 1.25-5 mg per image sequence, with additional doses permitted intraoperatively, provided the cumulative dose does not exceed 2 mg/kg [16]. Reported adverse reactions include hypersensitivity reactions, particularly urticaria and anaphylaxis; therefore, ICG should be avoided in patients with known hypersensitivity to ICG, and awareness of its transient interference with radioactive iodine uptake studies is also important. In the present case, the cumulative administered dose was 25 mg, remaining below the recommended maximum total dose.

## Conclusions

Mesenteric lymphangioma should be considered in the differential diagnosis of a large cystic mesenteric lesion in adults presenting with nonspecific abdominal symptoms or acute abdominal pain. Symptomatic lesions are best managed with complete surgical excision. This case demonstrates that robotic-assisted resection with adjunctive ICG fluorescence imaging can enable safe, organ-preserving excision of a giant mesenteric lymphangioma in a selected adult patient. Additional reports are needed to better define the role of robotics and fluorescence guidance in this rare clinical setting.
